# Shear bond strength and microleakage results for three experimental self-etching primer compositions for pit and fissure sealing

**DOI:** 10.1007/s00784-016-1907-z

**Published:** 2016-07-19

**Authors:** Sebastian Birlbauer, Meng-Ling Chiang, Christoph Schuldt, Vinay Pitchika, Reinhard Hickel, Nicoleta Ilie, Jan Kühnisch

**Affiliations:** 1 Department of Conservative Dentistry and Periodontology, Ludwig-Maximilians-University of Munich, Poliklinik für Zahnerhaltung und Parodontologie, Goethestraße 70, 80336 Munich, Germany; 2 Department of Pediatric Dentistry, Chang Gung Memorial Hospital, Taipei, Taiwan

**Keywords:** Pit and fissure sealant, Shear bond strength, Microleakage

## Abstract

**Objectives:**

This in vitro study evaluated the shear bond strength (SBS) and microleakage of three experimental self-etching primers for pit and fissure sealing.

**Materials and methods:**

The material used three formulations of an experimental fissure primer (EFP) applied without phosphoric acid etching (EFP-1/EFP-2/EFP-3) and one control group with sealant application after 30 s of acid etching. Four groups of sealants (*n* = 40 specimens/group) were tested for SBS, and a failure analysis was conducted after 1-day water storage, 3-month water storage, and 5000-fold thermocycling. In addition, microleakage was tested.

**Results:**

The SBSs of the EFPs (range 8.2 MPa (standard deviation 4.2) to 15.4 MPa (5.4)) were generally significantly lower than those of conventional fissure sealing (range 15.6 MPa (4.4) to 19.1 MPa (6.2)). The SBS of EFP-3 was better than that of the EFP-1 or EFP-2 formulations. Microleakage was significantly lower in the control group (1.1 %) than in the EFP-1 (3.8 %) and lower than in EFP-3 (7.7 %) group. In the (multiple) linear regression analysis, material and aging significantly influenced SBS.

**Conclusions:**

The SBS of EFP-3 was 15 to 32 % lower than it was for the corresponding controls.

**Clinical relevance:**

The SBS is lower, but the main potential benefit of this new approach is a reduced application time in clinical practice.

## Introduction

Pits and fissures are the most susceptible sites to caries in permanent teeth [1–3]. Pit and fissure sealants are frequently used to prevent caries development or to arrest existing caries lesions [4]. The effectiveness of sealants is obvious in patients at risk for caries [5], and this preventive measure protects pits and fissures as long as the sealant is fully retained on caries-susceptible sites [6]. Therefore, the clinical survival of sealant materials is an important prerequisite that might be predicted by assessing the shear bond strength (SBS). A large number of products of the same generic composition have been tested using in vitro studies. Methacrylate-based materials that showed acceptable long-term retention rates in in vivo studies [3] are considered to be easy to handle and are frequently used in clinical pratice; thus, these are the materials of choice for patients who are treated in a professional dental practice. New groups of sealants are under development, with the aim of simplifying and shortening the clinical workflow. Taking into account all treatment steps for self-etching primers and conventional sealants, the pretreatment time is reduced by up to 50 %. This might allow for a less technique-sensitive and more patient-friendly clinical workflow, which is particularly attractive in pediatric dentistry. Although the conventional clinical workflow employs a phosphoric acid etching step to create micromechanical retention, the new sealants use a self-etching primer to establish adhesion between the enamel and the sealant material [7, 8]. Several studies have been performed with and shown the potential of self-etching primers [9–16]. In this study, a new, self-etching, experimental fissure primer (EFP) was tested using three different formulas at three different time points to determine the SBS according to different aging procedures. Microleakage was also investigated. The EFP was referenced against conventionally sealed specimens using conventional 37 % phosphoric acid conditioning. The null hypothesis was that there is no difference between the tested EFPs and the control group.

## Material and methods

**Fig. 1:**
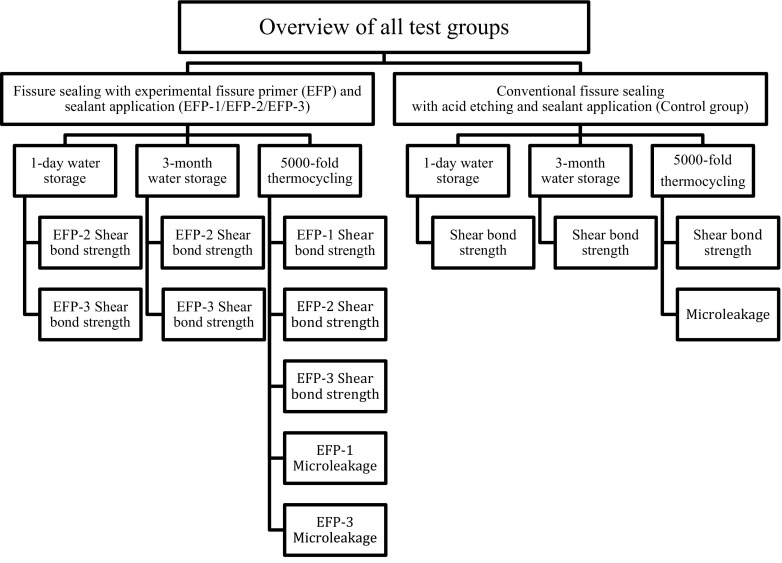
Overview of the investigated sealant procedures and the applied aging methods

**Fig. 2:**
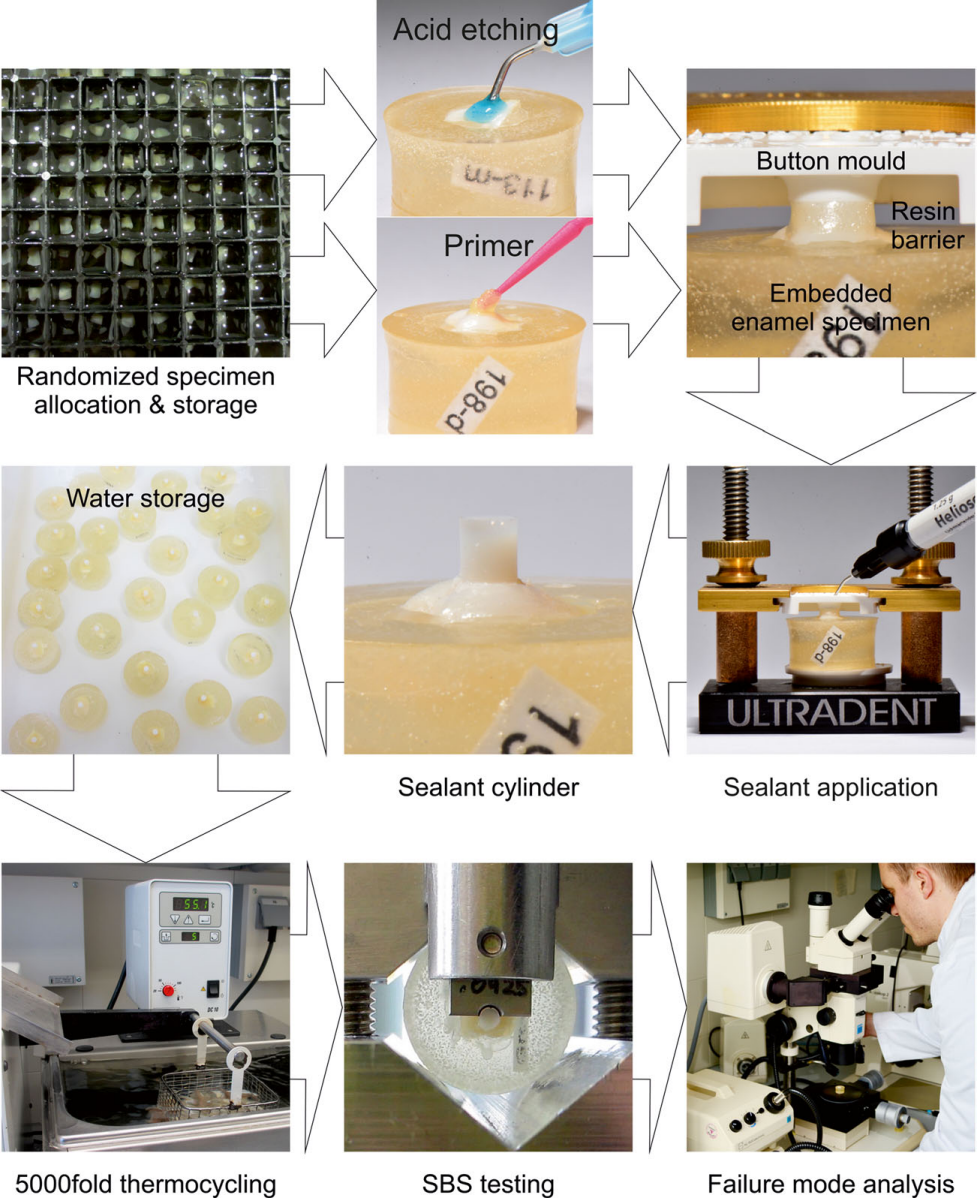
Illustration of the laboratory workflow

This in vitro study compared the SBS and microleakage of three experimental, self-etching primer compositions for pit and fissure sealing with those of controls. In addition, each material was aged according to three different protocols (Fig. 1). The laboratory workflow is summarized in Fig. 2.

### Specimen preparation for shear bond strength analysis

This study used 100 healthy, caries-free, extracted human third molars. The teeth were free of development disorders, fillings, and fissure sealing and showed complete root development. After extraction, the teeth were stored in sodium azide solution (0.2 %). Before being used for this study, all teeth were cleaned to remove adherent tissue and debris. All roots were removed by sectioning 1 mm under the cement-enamel junction, and the crowns were sectioned into four surfaces (mesial, distal, oral, buccal) with a diamond disk (Dental Diamond Disc, H 340-F-300, HORICO, Berlin, Germany). This process resulted in 400 tooth surfaces that were randomly assigned to each group (*n* = 40/10 from the mesial, distal, oral, and buccal surfaces) and stored in distilled water (pH ∼6.8, changing interval of 1 week; Fig. 2). Only one surface from each tooth was assigned to a group. All tooth surfaces were embedded in cold-curing methyl methacrylate resin (Technovit 4004, Heraeus Kulzer, Wehrheim, Germany). Each tooth surface was firmly aligned horizontally in the embedding material. All specimens were numbered according to a randomization table. After embedding, all tooth surfaces were cleaned with a rotary brush and fluoride-free polishing paste (Zircate Prophy Paste, Dentsply De Trey, York, PA, USA) and rinsed with a water spray. To simulate the clinical situation of fissure sealing, only unprepared specimens (aprismatic enamel) were used in this study.

### Experimental groups

Three different formulations of an experimental self-etching primer have been used (EFP, Ivoclar Vivadent, AG, Schaan, Liechtenstein; first formulation in 2011: LOT R52-074-1 (EFP-1); second formulation in 2013: LOT B61-139-1 (EFP-2); third formulation in 2014: LOT FA-219381 (EFP-3)). The formulations differed only in monomer composition; identical initiators and solvents were employed, as shown in Table 1.

**Table 1:** Composition of the experimental fissure primers (EFPs)

Lot	Monomers	Initiators	Solvents
EFP-1 R52-074-1	Bisacrylamide, bisacrylamide phosphate, methacrylate phosphonate	Bisacyl germanium, acyl phosphine oxide	Water, iso-propanol
EFP-2 B61-139-1	Bisacrylamide, bisacrylamide phosphate, methacrylate phosphonate, methacrylate crosslinker	Bisacyl germanium, acyl phosphine oxide	Water, iso-propanol
EFP-3 FA-219381	Bisacrylamide, methacrylate phosphate, hydrophilic methacrylate, methacrylate crosslinker	Bisacyl germanium, acyl phosphine oxide	Water, iso-propanol

The clinical application routine for fissure sealants was modified to allow for in vitro SBS tests. The primer was applied to the carefully cleaned enamel surface and agitated for 15 s with a microbrush (EFP-1: microbrush does not bend; EFP-2 and EFP-3: microbrush bends during application). The resulting primer coat was then carefully dried with pressurized air for 5 to 8 s to achieve a thin film. The teeth were then inserted into the sample jig (ISO 29022, Ultradent Products, South Jordan, UT, USA), and the cylindrical plastic mold (Button Mold Insert, ISO 29022, Ultradent Products, Inc., South Jordan, UT, USA) was lowered to achieve a gap-free fit on the primer-treated enamel surface. The fissure sealant material (Helioseal F, Ivoclar Vivadent, Schaan, Liechtenstein, EFP-1: LOT P21374, EFP-2: LOT S10257, EFP-3: LOT S03724) was then applied in two steps. Each layer was light cured for 20 s with a light curing unit (Bluephase Style, 1200 mW/cm^2^, wavelength 385–515 nm, Ivoclar Vivadent, Schaan, Liechtenstein). To prevent uncontrolled leakage of the fissure sealing material onto the enamel, a light-cured resin barrier (OpalDam, Ultradent Products, South Jordan, UT, USA) was applied around the perimeter of the plastic mold before applying the fissure sealant material. After light curing the second application of the fissure sealant, the resin barrier and plastic mold were carefully removed. The procedure rendered a composite cylinder with a 2.37 mm diameter perpendicular to the enamel surface, as required by ISO 29022(2013).

### Control group—conventional fissure sealing

All enamel surfaces were initially rinsed with a water spray and dried with water- and oil-free air, followed by etching with 37 % phosphoric acid gel (Total Etch, Ivoclar Vivadent, Schaan, Liechtenstein) for 30 s. The tooth surface was then rinsed with a water spray and dried with pressurized air for 5 s until a chalky-white enamel surface was visible. The fissure sealing material (Helioseal F, Ivoclar Vivadent, Schaan, Liechtenstein; LOT numbers provided above) was then applied as described above.

### Specimen aging

Three different aging procedures were used to simulate the influence of the oral environment as follows: (1) 1-day storage in distilled water at 37 °C (Jouan EU3, INNOVENS Ovens, ThermoFisher Scientific Inc., Waltham, MA, USA); (2) 3-month storage in distilled water at 37 °C that was changed at 1-week intervals; and (3) 1-day storage in distilled water at 37 °C, followed by thermocycling (Haake W15, Thermo Haake GmbH, Karlsruhe, Germany) between 5 (±2) and 55 °C (±2) for 5000 cycles, with a dwell time of 30 s and a transfer time of 5 s. The overall protocol is shown in Fig. 1.

### Shear bond strength

All cylindric test specimens were checked for proper configuration and quality. No deficits after treatment, e.g., air bubbles and/or sealant spillage, and no initial loss of sealant cylinders prior to SBS testing was observed. A notched-edge SBS test (Ultradent, ISO 29022) was performed for the EFP-2 and EFP-3 groups and the control group. The EFP-1 group was tested using the Guillotine method employing a flat knife edge blade, as was the standard practice in 2011. After sample aging, the SBS test was performed in a universal testing machine (MCE 2000ST, Quicktest Prüfpartner GmbH, Langenfeld, Germany) at a crosshead speed of 1 mm per minute. Specimens were aligned in a metal sample holder (test base clamp, ISO 29022, Ultradent Products, South Jordan, UT, USA) with the occlusal tooth surface facing down (“crown down”). The notched-edge shear fixture (notched-edge crosshead assembly, ISO 29022, Ultradent Products, South Jordan, UT, USA) with the shear blade (notched-edge shear blade, ISO 29022, Ultradent Products, South Jordan, UT, USA) was mounted to the universal testing machine and placed over the composite cylinder on the aligned specimen. After precise positioning of the shear blade over the composite cylinder, the load was applied at a constant crosshead speed of 1 mm/min until the material failed. The maximum force (N) up to failure was recorded. The SBS (expressed in MPa) was calculated from the maximum force and the bonded area of the fissure sealant on the tooth surface. All specimens were also examined for failure modes using a stereomicroscope (Stemi SV11, Carl Zeiss AG, Jena, Germany) at 20-fold magnification. Failures were classified as (1) adhesive failure, (2) cohesive failure within material, (3) mixed failure (adhesive and cohesive within material), and (4) enamel failure.

### Microleakage

Using teeth stored and cleaned as previously described, 36 human molars were assigned to investigate microleakage for the EFP-1 (*n* = 20), EFP-3 (*n* = 8), and control (*n* = 8) groups. Sealing using the natural fissure pattern was then performed in strict accordance with the manufacturer’s instructions (see above). All specimens were stored in distilled water at 37 °C for 24 h in a heating oven (Jouan EU3, INNOVENS Ovens, ThermoFisher Scientific Inc., Waltham, MA, USA). The specimens were then aged by thermocycling, as described above. After thermocycling, the root surfaces were isolated with tacky wax (Boxing Wax Sticks, Kerr Corporation, Romulus, MI, USA). To avoid dye penetration to other parts of the teeth, the entire surface of each tooth, except the area within 1 mm of the fissure sealant, was then covered with two coats of nail varnish. The specimens were then immersed in 0.5 % basic fuchsine solution for 24 h at 37 °C. Then, all specimens were rinsed with copious amounts of water, and the roots were sectioned off 1 mm under the cement-enamel junction with a diamond disk (Dental Diamond Disc, H 340-F-300, HORICO, Berlin, Germany). The tooth crowns were then fully embedded in cold-curing methyl methacrylate resin (Technovit 4004, Heraeus Kulzer GmbH, Wehrheim, Germany). This treatment resulted in a rectangular block of approximately 2.5 × 1.2 × 0.8 cm^3^ for each tooth. The blocks were fixed in a sectioning saw (Isomet Low Speed Saw, Buehler, Lake Bluff, IL, USA) with a diamond blade (Diamond Blade, Leco, St. Joseph, MI, USA), and the crowns were sectioned in a buccolingual direction into at least five 1-mm-thick slices. The front and back of each slice were used for inspection, which led to at least 10 analyzed surfaces per tooth. Dye penetration was analyzed using a stereomicroscope (Stemi SV11, Carl Zeiss AG, Jena, Germany) at 20-fold magnification. Each slice surface was photographed with a digital single lens reflex camera, and possible quality loss such as dye penetration at sealant fractures, sealant detachment, and fissure sealant defects was recorded. Each dye-penetrated sealant section was quantitatively measured, and the measurement was expressed as a percentage of the length of dye penetration in relation to the total length of the sealant on enamel; thin films of primer or sealant were not considered. All measurements were performed with ImageJ software (Version 1.47, Wayne Rasband, National Institutes of Health, Bethesda, MD, USA). Dye penetration was not rated as microleakage if it occurred visibly through the enamel and/or dentine cracks along the cement-enamel junction or through fissure sealant cracks.

### Statistics

A formal sample size calculation was performed using 95 % confidence level, 80 % power, and a two-tailed significance level of 5 %. The expected mean difference in the SBS between the two groups would be 3 MPa with a variance of 20 and 25 for two groups, respectively. This resulted in a sample size of 40 specimens per group (10 pieces each from mesial, distal, buccal, and lingual). In addition, to minimize the influence of one tooth on the results, the pieces were randomized so that no tooth’s piece will be used more than once in a group. The descriptive and explorative data analysis was performed using SPSS Statistics for Windows, Version 22.0 (SPSS Inc., an IBM Company, Chicago, USA). Descriptive statistics for SBS and microleakage were calculated and presented as the mean and standard deviation or as percentages, respectively. The Shapiro-Wilk test was used to test whether the SBS, and microleakage data were normally distributed; the data did not have a Gaussian distribution. Pairwise comparisons were made with respect to the material and the technique employed using the Mann-Whitney *U* test. Multiple linear regression analysis was performed to predict the influence of the material and aging technique on the SBS in model 1. Model 2 was constructed by including the interaction term (material and aging technique together) to evaluate any significant effect. A two-tailed α significance level of 0.05 and a confidence level of 95 % were used for all analyses.

## Results

This study showed that EFPs were associated with lower SBS (Table 2) and microleakage (Table 5) when compared to the controls, which used a conventional fissure sealing. According to the SBS data, the EFP-3 group showed the highest values under in vitro conditions compared with the groups that were tested earlier. Nevertheless, the SBS was generally significantly lower compared with the control group (1 day of water storage 21 %; 3 months of water storage 15 %; 5000-fold thermocycling 32 %). The lowest SBS values were documented after 5000 thermocycles.

**Table 2:** Shear bond strength of the tested sealant procedures

Shear bond strength (MPa)	Mean (standard deviation) min-max		
	1-day water storage	3-month water storage	5000-fold thermocycling
EFP-1	Not performed	Not performed	8.2 (4.2)B, C
			2.5–18.8
EFP-2	9.1 (3.5)D, E	8.7 (3.4)D, E	9.5 (5.4)D, E
	3.8–16.4	0.9–16.2	2.9–18.4
EFP-3	15.1 (5.0)D, F	15.4 (5.2)D	10.6 (2.4)B, D, F
	3.4–26.0	6.5–28.6	4.3–16.1
Control group—conventional fissure sealing with 30 s acid etching	19.1 (6.2)a, E, F	18.2 (7.5)E	15.6 (4.4)a, C, E, F
	8.8–35.3	5.1–30.3	6.1–22.3

Lowercase letters indicate a significant difference between the aging procedures (within the rows)

Uppercase letters indicate significant differences between the tested sealant in relation to the applied aging procedure (within the columns)

Mann-Whitney *U* test, *p* < 0.05

The failure mode analysis results are shown in Table 3. The most common failure types were adhesive fractures (67.5–90.0 %), followed by mixed failures (7.5–22.5 %). According to multiple linear regression analysis, both models 1 and 2 significantly influenced the SBS. Because the interaction term in model 2 had a significant effect on changing the SBS, therefore model 2 was considered to be an appropriate choice (Table 4). Multiple linear regression analysis predicted that the estimate of the reference was 19.15 and that EFP-2, EFP-3, 3-month water storage, and 5000-fold thermocycling significantly reduced the SBS (estimate of SBS = 9.09, 15.15, 18.15, and 15.61 MPa, respectively). Although model 2 significantly influenced the SBS, making the interaction term (material and aging together) an influencing factor, this effect was more pronounced for the EFP-2 group and for 5000-fold thermocycling and was not significant for the other variables. Table 5 presents the microleakage results. A significant difference was found only between the EFP-1 group and the control group; no such difference was found for the EFP-3 group.

**Table 3:** Failure mode analysis of the tested sealant procedures after measuring the shear bond strength

Failure mode analysis (*N*/%)	1-day water storage	3-month water storage	5000-fold thermocycling
EFP-1			
Adhesive failure	Not performed	Not performed	16/80.0
Cohesive failure			0/0.0
Mixed failure			4/20.0
Enamel failure			0/0.0
EFP-2			
Adhesive failure	36/90.0	36/90.0	36/90.0
Cohesive failure	0/0.0	1/2.5	0/0.0
Mixed failure	4/10.0	3/7.5	4/10.0
Enamel failure	0/0.0	0/0.0	0/0.0
EFP-3			
Adhesive failure	29/72.5	27/67.5	35/87.5
Cohesive failure	0/0.0	0/0.0	0/0.0
Mixed failure	10/25.0	11/27.5	4/10.0
Enamel failure	1/2.5	2/5.0	1/2.5
Control group			
Adhesive failure	31/77.5	33/82.5	32/80.0
Cohesive failure	0/0.0	0/0.0	0/0.0
Mixed failure	9/22.5	7/17.5	7/17.5
Enamel failure	0/0.0	0/0.0	1/2.5

**Table 4:** Multiple linear regression results presenting the estimates, standard errors, and corresponding *p* values, showing the influences of material, aging, and both together (interaction term) on the SBS of the EFP-2, EFP-3, and control group

	Multiple linear regression models			
Factor	Factor level	Estimate for the SBS in MPa	Standard error	*p* value
Model 1				
Reference value	–	18.62	0.60	–
Material	EFP-2	10.09	0.66	<0.001*
	EFP-3	14.71	0.66	
Aging	3-month water storage	18.24	0.66	<0.001*
	5000-fold thermocycling	16.05	0.66	
Model 2				
Reference value	–	19.15	0.80	–
Material	EFP-2	9.09	1.13	<0.001*
	EFP-3	15.15	1.13	
Aging	3-month water storage	18.15	1.13	<0.001*
	5000-fold thermocycling	15.61	1.13	
Material and aging	EFP-2 and 3-month water storage	19.77	1.59	0.003*
	EFP-3 and 3-month water storage	20.37	1.59	
	EFP-2 and 5000-fold thermocycling	23.15*	1.59	
	EFP-3 and 5000-fold thermocycling	18.08	1.59	

The control group with 1-day water storage served as reference value in relation for model 1 (material and aging, separately) and for model 2 (material, aging, and both together), respectively. Finally, the estimates from the linear regression analysis are representing the mean SBS (in MPa) for each stratum

*Significance according to multiple linear regression analysis (*p* < 0.05)

**Table 5:** Microleakage of the tested sealants following 5000-fold thermocycling

Microleakage	EFP-1	EFP-3	Control group
Number of teeth (N)	20	8	8
Number of all available tooth sides (N)	240 (100.0 %)	88 (100.0 %)	104 (100.0 %)
Number of sides with any quality loss (N)	62 (25.8 %)	27 (30.7 %)	21 (20.2 %)
Surfaces with dye penetration (N)	45	15	19
Surfaces with dye penetration at sealant fractures (N)	10	12	2
Detachment of fissure sealant (N)	6	–	–
Defects of fissure sealant (N)	1	–	–
Number of sides without any quality loss (N)	178 (74.2 %)	61 (69.3 %)	83 (79.8 %)
Mean microleakage (SD)	3.8 % (9.3)^a^	7.7 % (19.4)	1.1 % (3.9)^a^
Minimum	0 %	0 %	0 %
Maximum	59.3 %	82.3 %	36.1 %

Microleakage of the EFP-2 group was not investigated

^a^A significant difference between the compared sealant procedures, Mann-Whitney *U* Test, *p* < 0.05

## Discussion

The documented results of this study have to be considered in relation to its methodology. Relative to other investigations [17], this study included a large number of specimens per group (40 human tooth samples) that were randomly allocated. In addition, three different methods were applied for specimen aging to address the possible effects of thermal stress or hydrolytic instability. The combination of these test procedures provides an overview of the effects of different aging scenarios in relation to the SBS. In addition, the latest recommendations of ISO 29022 [18] were followed rather than the former standard, which recommended a flat-shear blade for the evaluation of the SBS [19]. As a result, the teeth in the earliest investigated EFP-1 group were sheared with a flat knife edge blade, which might limit the comparability of that group. Regardless, no significant differences between methods were found [20]. Another potential limitation might be that aprismatic enamel was used in this investigation, which represents the typical clinical situation for pit and fissure sealing. In contrast, the ISO standard requires prismatic enamel use. A challenge with this adaptation is that the slightly curved surfaces of non-prepped teeth may complicate the process of aligning the cylindrical plastic mold, possibly leading to sealant leakage from underneath the mold and consequently to variations in the bonded area. Here, two strategies to overcome this problem were followed. First, the chosen enamel area was as flat as possible. Secondly, a fluid resin barrier was applied to prevent the leakage of sealant material [10]. Although grinding the enamel surfaces would result in flat and reproducible surfaces, this would remove the clinically relevant aprismatic enamel layer to which fissure sealants are normally applied. In view of the small standard deviations for SBS (Table 2), the usage of natural enamel surfaces with an intact outer aprismatic layer seems to have a limited influence.

Another limiting factor of this study might be the inclusion of microleakage testing because it does not predict clinical performance, was evaluated as not reliable, and is difficult to repeat [17]. Regardless, microleakage testing has been used by several working groups to give a rough outline of the adhesive compound in sealant materials. This was also observed in the present study as the EFP showed a higher microleakage in comparison to the conventional fissure sealing. The main finding of this in vitro investigation was that the SBS of the newly developed experimental self-etching primer (e.g., 10.6 MPa/5000-fold thermocycling) was generally significantly lower than that of conventional fissure sealing procedures (e.g., 15.6 MPa/5000-fold thermocycling; Table 2). Microleakage testing showed heterogeneous results, with the EFP-1 group showing significantly greater dye-penetrated proportions compared to the control group. Therefore, the initially formulated hypothesis that there was no difference between the procedures was rejected. This finding can be linked to the fact that the adhesive pretreatment and application routines of the EFPs are inferior to those of conventional acid etching. The latter technique removes the outer prismless enamel layer, exposes the prisms, and enlarges the surface area, which is associated with a microretentive pattern that enables a perfect bond between the enamel and the sealant. EFPs do not remove the aprismatic layer and initiate a chemical compound between adhesive and enamel, which can explain the divergent results. Therefore, the achievement of a robust bond between aprismatic enamel and EFPs remains a challenge. Nevertheless, it should be noted that improvements in the formulation of the EFPs have been made over time, but the reference standard of acid etching has not been fully achieved (Table 2).

When the SBS results of this study are compared with previously published data, the following pattern for other self-etching primers was observed. In some investigations, a similar order of magnitude for the SBS was found, such as Adper Prompt L-Pop (10.8 MPa after 500-fold thermocycling [9]; 15.8 MPa after 1 week of water storage [10]; 13.9 MPa after 1 year of water storage [10], and 12.9 MPa after 500-fold thermocycling [11]); Adper Single Bond 2 (12.08 MPa after 1 day of water storage [12]); Admira Bond (7.9 MPa after 500-fold thermocycling [13]); Gluma Primer (12.3 MPa after 200-fold thermocycling [14]); and Scotchbond 2 (12.5 MPa after 200-fold thermocycling [14]).

It is important to note that some studies have shown higher SBS values for self-etching primers, such as Scotchbond Multi-Purpose plus Alpha Seal (30.7 MPa after 1 day of water storage [15]); Scotchbond Multi-Purpose plus Fluoroshield (18.7 MPa after 1 day of water storage [15]); XenoIII plus Eco-S (19.7 MPa after 1 day of water storage [16]); and XenoIII plus Cilinpro (20.8 MPa after 1 day of water storage [16]). Importantly, some author groups pretreated the enamel with phosphoric acid before applying the self-etching primer, which limits the comparability [16]. In addition, the reduced phosphoric etching contact time in the control groups might lead to a less-pronounced microretentive etching pattern and a comparatively reduced SBS in control groups [15, 16]. With respect to the methodological details of the aforementioned studies, it should be noted that aging protocols, particularly the length of water storage and the number of thermocycles, differ significantly throughout the in vitro protocols and may also influence the results. This influence was clearly shown in the results of the linear regression analysis (Table 4). Here, both the aging technique and the material significantly influenced the SBS. Therefore, the documented SBS values and trends should not be discussed independent of each study’s methodology. This fact may also indicate a harmonization of the testing protocols for SBS studies on fissure sealants with the aim of increasing the comparability among studies.

Finally, the potential of the new materials for clinical dental practice should be discussed. On the one hand, it should be mentioned that the SBS for the EFP was significantly lower compared to acid etching pretreatment, and therefore, the long-term sealant retention rate might also decrease under clinical conditions. However, this hypothesis has to be proven in a clinical study before a new product can be recommended for daily dental practice. On the other hand, the approach used to establish a chemical bond between aprismatic enamel and adhesive, while excluding conventional acid etching, should be highlighted. The main advantage of this technique is a shorter and simplified clinical workflow, which is of particular importance for pediatric patients. With respect to the promising SBS and microleakage values for the EFP formulations, it is possible that future improvements may increase the material performance.

## Conclusion

Within the limitations of the present study, it could be concluded that all tested versions of the experimental self-etching primer in combination with a pit and fissure sealant generally resulted in significantly lower SBS and microleakage compared with conventional phosphoric acid etching. Regardless, the primer showed encouraging results on aprismatic enamel, and these results should be verified under clinical conditions in a future study. In addition to the significant influence of the material, the logistic regression analysis revealed a significant influence of the aging procedure.

## References

[1] Manton DJ, Messer LB (1995). Pit and fissure sealants: another major cornerstone in preventive dentistry. Aust Dent J.

[2] Hannigan A, O’Mullane DM, Barry D, Schäfer F, Roberts AJ (2000). A caries susceptibility classification of tooth surfaces by survival time. Caries Res.

[3] Kühnisch J, Mansmann U, Heinrich-Weltzien R, Hickel R (2012). Longevity of materials for pit and fissure sealing—results from a meta-analysis. Dent Mater.

[4] Welbury R, Raadal M, Lygidakis NA (2004). EAPD guidelines for the use of pits and fissure sealants. Eur J Pediatr Dent.

[5] Ahovou-Saloranta A, Hiiri A, Nordblad A, Mäkelä M, Worthington HV (2008). Pit and fissure sealants for preventing dental decay in the permanent teeth of children and adolescents. Cochrane Database Syst Rev.

[6] National Institutes of Health Concensus Development conference Statement (1984). Dental sealants in the prevention of tooth decay. J Dent Educ.

[7] Jain P, Stewart GP (2000). Effect of dentin primer on shear bond strength of composite resin to moist and dry enamel. Oper Dent.

[8] Woronko GA, St Germain HA, Meiers JC (1996). Effect of dentin primer on the shear bond strength between composite resin and enamel. Oper Dent.

[9] Asselin ME, Sitbon Y, Fortin D, Abelardo L, Rompre PH (2009). Bond strength of a sealant to permanent enamel: evaluation of 3 application protocols. Pediatr Dent.

[10] Peutzfeldt A, Nielsen LA (2004). Bond strength of a sealant to primary and permanent enamel: phosphoric acid versus self-etching adhesive. Pediatr Dent.

[11] Biria M, Ghasemi A, Torabzadeh H, Shisheeian A, Baghban AA (2014). Assessment of microshear bond strength: self-etching sealant versus conventional sealant. J Dent (Tehran).

[12] Borsatto MC, Thomaz MY, Contente MM, Gomes-Silva JM, Mellara JM, Tde S, Galo R, Palma-Dibb RG (2010). Bonding agent underneath sealant: shear bond strength to oil-contaminated enamel. Braz Dent J.

[13] Eminkahyagil N, Gokalp S, Korkmaz Y, Baseren M, Karabulut E (2005). Sealant and composite bond strength to enamel with antibacterial/self-etching adhesives. Int J Paediatr Dent.

[14] Garcia-Godoy F, Cooley RL, Ranly DM, Burger KM (1991). Effect of dentin adhesives on sealant bond strength. J Clin Pediatr Dent.

[15] Souza-Junior EJ, Borges BC, Montes MA, Alonso RC, Ambrosano GM, Sinhoreti MA (2012). Influence of etching time and bonding strategies on the microshear bond strength of self- and light-cured pit-and-fissure sealants. Braz Dent J.

[16] Dhillon JK, Pathak A (2012). Comparative evaluation of shear bond strength of three pit and fissure sealants using conventional etch or self-etching primer. J Indian Soc Pedod Prev Dent.

[17] Heintze SD, Zimmerli B (2011). Relevance of in vitro tests of adhesive and composite dental materials. A review in 3 parts. Part 3: in vitro tests of adhesive systems. Schweiz Monatsschr Zahnmed.

[18] International Organisation for Standardisation ISO 29022:2013. Dentistry-adhesion-notched-edge shear bond strength test

[19] Braga RR, Meira JB, Boaro LC, Xavier TA (2010). Adhesion to tooth structure: a critical review of “macro” test methods. Dent Mater.

[20] Pitchika V, Chiang ML, Birlbauer S, Crispin A, Hickel R, Ilie N, Kühnisch J (2015). Which methodological factors influence the shear-bond strength of fissure sealants?. Clin Oral Investig.

